# *H. pylori* Seropositivity before Age 40 and Subsequent Risk of Stomach Cancer: A Glimpse of the True Relationship?

**DOI:** 10.1371/journal.pone.0017404

**Published:** 2011-03-02

**Authors:** Christina Persson, Yanbin Jia, Helena Pettersson, Joakim Dillner, Olof Nyrén, Weimin Ye

**Affiliations:** 1 Department of Medical Epidemiology and Biostatistics, Karolinska Institutet, Stockholm, Sweden; 2 Swedish Institute for Infectious Disease Control, Solna, Sweden; 3 Department of Laboratory Medicine, Karolinska Institutet, Stockholm, Sweden; Statens Serum Institute, Denmark

## Abstract

Stomach carcinogenesis involves mucosal and luminal changes that favor spontaneous disappearance of *Helicobacter pylori*. Therefore, the association between the infection and cancer risk might typically be underestimated. As acquisition of the infection almost invariably occurs before adulthood, the serostatus at age 16–40 should best reflect the lifetime occurrence of the infection. We therefore conducted a case-control study nested within a historic cohort of about 400,000 individuals who donated sera before age 40 to either of two large Swedish Biobanks between 1968 and 2006, and whose records were linked to complete nationwide registers. For each stomach adenocarcinoma case occurring at least 5 years after serum donation 2 controls were selected matched on age, sex and year of donation and biobank. Serum immunoglobulin G antibodies against *H. pylori* cell-surface antigens (Hp-CSAs) were measured with an enzyme–linked immunosorbent assay and antibodies against CagA with an immunoblot assay. Conditional logistic regression models were used to estimate odds ratios (ORs) for stomach adenocarcinoma among *H. pylori* infected relative to uninfected. We confirmed 59 incident cases of stomach adenocarcinoma (41 non-cardia tumors) during follow-up. ORs for non-cardia stomach adenocarcinoma among subjects with Hp-CSA antibodies (regardless of CagA serostatus), antibodies against CagA (regardless of Hp-CSA serostatus), and antibodies to both, relative to those who were seronegative to both, were 17.1 (95% confidence interval [CI] 4.0–72.9), 10.9 (95% CI 3.2–36.9), and 48.5 (95% CI 5.8–407.4), respectively. *H. pylori* infection is a much stronger risk factor for non-cardia stomach adenocarcinoma than initially realized. However, further studies are needed to answer whether it is a necessary cause, as the possibility of misclassification of *H. pylori* status could not be ruled out in our study.

## Introduction

Although the incidence of stomach adenocarcinoma has declined in the industrialized world, most likely due to the declining rates of *Helicobacter pylori* (*H. pylori)* infections, it is still the fourth most common type of cancer, and due to the poor prognosis the second most common cause of cancer-related deaths worldwide [1], [2]. Notwithstanding its epithet as the by far strongest established risk factor for distal stomach cancer [3], *H. pylori* infection has remained a dark horse in regard to its true attributable fraction, in turn determined by the true strength of the association. Serological tests of antibodies against *H. pylori* cell surface antigens (Hp-CSAs) and CagA encoded by cytotoxin-associated gene-A, known to cause more extensive inflammation in the stomach mucosa, have long been employed to explore this association. However, chronic atrophic gastritis, occurring at a rate of 0.9–3.6 per 100 person-years among untreated infected individuals [4] and probably at a much higher rate among those predestined to develop stomach cancer, destroys the microorganism's natural niche and may lead to disappearance of *H. pylori* and decreasing antibody titres. This might have resulted in underestimation of the strength of the association in previous studies. To measure *H. pylori* infection status in early adulthood till 40 years of age, when essentially all infected people have already acquired their infection [5] but chronic atrophic gastritis is still rare [6], would be critical for accurately measuring the true association. We therefore conducted a case-control study nested within a historic cohort of about 400 000 individuals who donated serum samples to either of two large Swedish Biobanks at young ages between 1968 and 2006 to estimate the true strength of the *H. pylori*-stomach cancer relationship.

## Methods

The cohort included all individuals who donated serum before 40 years of age to either of the two Swedish Biobanks – the Swedish Institute for Infectious Disease Control Biobank (since 1957) and Malmö Microbiology Biobank (since 1969).

### Study biobanks

#### Swedish Institute for Infectious Disease Control Biobank

The Swedish Institute for Infectious Disease Control has performed a series of population-based, nationwide investigations of the immunity against infections in the Swedish population [7]. For decades, Swedish Institute for Infectious Disease Control has also served as reference laboratory for many microbiological analyses. As a part of the quality control and documentation system, part of the samples that have been analyzed have been stored at −20°C. Samples have been stored since 1957 and complete series of samples are stored since 1977. The biobank included serum samples from 93 462 unique individuals (with valid National Registration Numbers [NRNs] – individually unique personal identifiers assigned to all Swedish residents shortly after birth or immigration) collected between age 16 and 40. The samples selected for the study cohort arrived in the biobank between 1968-03-19 and 2001-12-31.

#### Malmö Microbiology Biobank

The biobank contains the samples submitted to the Department of Clinical Microbiology at MAS University Hospital in Malmö, where saving of a part of the sample has been necessary for clinical diagnostic and documentation purpose. The majority of the samples are serum samples that have been submitted for diagnosis of blood-borne viral infections, e.g. hepatitis virus, (over 500,000 samples). About 100,000 samples in the biobank emanate from population-based serological screening for viral infections and rubella immunity during pregnancy. The population served is currently the entire region of Skåne, where the Department of Clinical Microbiology is currently the only clinical virological laboratory. The operation of the biobank is certified by the Swedish National Accreditation Agency and follows ISO17025 procedures. The Malmö Microbiology Biobank included serum samples from 374 598 unique individuals (with valid NRNs) collected between age 16 and 40. The samples selected for the study cohort arrived in the biobank between 1969-02-10 and 2001-12-31.

### Follow-up

Linkage to complete nationwide registers, including the Swedish Cancer Register, Causes of Death Register and Migration Register, provided follow-up data from serum collection until first diagnosis of stomach cancer, emigration, death, or December 31, 2006, whichever occurred first. The NRNs, used both in the biobanks and the registers, ensured precise matches. To verify the validity of the NRNs, further linkage to the Total Population Register was performed. If a NRN could not be found in the Total Population Register, Causes of Death Register or Migration Register, it was deemed to be invalid and the serum sample, along with the record, was not included in our analysis. Stomach adenocarcinoma cases were identified through the nationwide Cancer Register, founded in 1958 and with an estimated completeness of 96–98% [8], [9]. The Cancer Register recorded neoplasms using ICD-7 classification through the entire study period. A separate code for gastric cancer in the cardia was introduced first in 1969 and was widely used since 1970. The histological type of each recorded tumour was also recorded by a histology code, albeit these codes did not distinguish between Laurén's intestinal and diffuse types of adenocarcinoma [10]. In order to reduce the possibility of misclassification of *H. pylori* infection status associated with stomach cancer development, stomach cancer cases occurring within 5 years of index serum collection were disregarded. For each stomach cancer case we selected 2 controls matched on age, sex, year of serum sample collection and biobank using incidence density sampling.

### Review of medical records

To further verify the diagnosis and specify the gastric subsite, all medical records collected from corresponding treatment units were reviewed by a specialist in gastrointestinal surgery (ON) blinded to *H. pylori* status and earlier reported ICD code in the Swedish Cancer Register.

### Laboratory analysis

Immunoglobulin G antibodies against Hp-CSAs were measured with an enzyme–linked immunosorbent assay (ELISA) (Biohit, Finland). The sensitivity and specificity for the assay was reported as 96% and 94%, respectively [11]. Antibodies against CagA were measured with an immunoblot assay (Helicoblot 2.1, Genelabs Diagnostics, Singapore) according to manufacturer's instructions [12].

Pepsinogen (PG) levels in sera were measured using an ELISA method (Biohit, Finland) according to the manufacturer's instructions. Presence of severe or moderate corpus atrophic gastritis was defined as PGI below 25 ug/l or PGI:PGII ratio less than 3. The sensitivity and specificity was reported as 71% and 98% in a validation study performed in Sweden [13]. All laboratory analyses were carried out in a blinded fashion by trained personnel. The quality control samples provided with the kits were included on each assay plates with additional quality control samples using pooled serum samples from the laboratory.

### Statistical analysis

The odds ratios (ORs) and their corresponding 95% confidence intervals (95% CIs) of developing stomach adenocarcinoma associated with *H. pylori* infection was estimated using conditional logistic regression models. Since controls were individually matched to the cases with regard to age, sex, calendar year of serum collection, and biobank, these factors were inherently adjusted for. All statistical analyses were performed using Stata/SE 9.2 (StataCorp, college Station, TX, USA). The study was approved by the Regional Research Ethics Vetting Board in Stockholm, Sweden.

## Results

**Figure 1:**
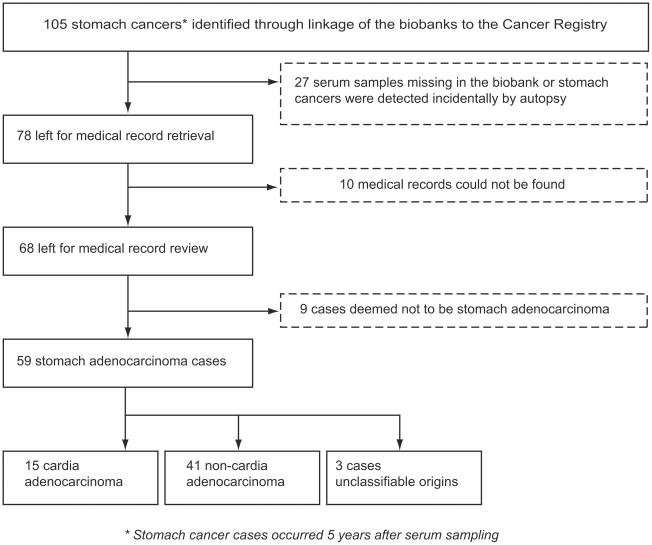
Flowchart for identifying stomach adenocarcinoma cases. In total 105 stomach cancers (ICD-7 code 151) which occurred at least 5 years after blood sample collection were identified during follow-up. Among these, 27 were excluded due to either missing serum samples or the fact that the cancers were found incidentally at autopsy. For the remaining 78 cases, medical records related to cancer diagnosis for 68 cases were successfully retrieved. After review, 9 cases were deemed to be tumors other than stomach adenocarcinoma. Among the remaining 59 stomach adenocarcinoma cases, 15 stomach adenocarcinoma cases were determined to be cardia adenocarcinoma and 41 as non-cardia stomach adenocarcinoma. In 3 stomach adenocarcinoma cases it was not possible to determine the exact origin in the stomach.

**Figure 1** shows the flowchart for identification of stomach adenocarcinoma cases. In total 105 stomach cancers (ICD-7 code 151) which occurred at least 5 years after blood sample collection were identified during follow-up. Among these, 27 were excluded due to either missing serum samples or the fact that the cancers were found incidentally at autopsy. For the remaining 78 cases, we successfully retrieved medical records related to cancer diagnosis for 68 cases. After review, 9 cases were deemed to be tumors other than stomach adenocarcinoma. Among the remaining 59 stomach adenocarcinoma cases, 15 stomach adenocarcinoma cases were determined to be cardia adenocarcinoma and 41 as non-cardia stomach adenocarcinoma. In 3 stomach adenocarcinoma cases it was not possible to determine the exact origin in the stomach.

Some basic characteristics of cases and their matched controls are summarized in **Table 1**. As expected, there were no differences in the sex distribution and mean age at blood sample collection between cases and controls. The mean age at diagnosis of stomach adenocarcinoma was 47.3 years (range 25–68). The mean time between serum collection and stomach adenocarcinoma diagnosis was 16.5 years (range 5–33). Presence of severe or moderate corpus atrophic gastritis at time of initial serum collection was more common among stomach adenocarcinoma cases (8.5%) as compared with matched controls (0.9%).

**Table 1:** Characteristics of the stomach adenocarcinoma cases and their matched controls.

Characteristics	Stomach adenocarcinoma cases (n = 59)	Controls (n = 117)
Men (%)	30 (50.9)	60 (51.2)
Age at sample collection, mean (SD), range	30.8 (6.1), 16–40	30.9 (6.0), 16–40
Age at cancer diagnosis, mean (SD), range	47.3 (9.4), 25–68	
Years between serum collection and cancer diagnosis, mean (SD), range	16.5 (7.2), 5–33	
Severe or moderate corpus atrophy*, N (%)	5 (8.5)	1 (0.9)

* Defined as serum pepsinogen I<25 ug/l or pepsinogen I:II ratio <3 at time of initial serum collection.

ORs for all stomach adenocarcinoma among subjects with Hp-CSA antibodies (regardless of CagA serostatus) and antibodies against CagA (regardless of Hp-CSA serostatus) were 4.1 (95% CI 1.9–8.5) and 3.5 (95% CI 1.7–7.1), respectively (**Table 2**). We further analyzed data by combining Hp-CSA and CagA antibodies, and used subjects seronegative for both as the unexposed reference group. Compared with this ‘clean’ reference group, those seropositive with either Hp-CSA or CagA antibodies had a more than 3-fold increased risk of developing stomach adenocaricnoma. The increased risk was more pronounced among the group seropositive with both Hp-CSA and CagA antibodies (OR = 5.5, 95% CI 2.3–12.9) (**Table 2**).

**Table 2:** *H. pylori* -CSAs and CagA seropositivity and the risk of stomach adenocarcinoma overall, cardia site and non-cardia site.

		Stomach adenocarcinoma			Cardia adenocarcinoma			Non-cardia adenocarcinoma		
Serological test results		Case	Control	OR*(95% CI)	Case	Control	OR*(95% CI)	Case	Control	OR*(95% CI)
Hp-CSAs antibodies	Negative	20	76	Ref.	12	21	Ref.	6	51	Ref.
	Positive	39	41	4.1 (1.9–8.5)	3	9	0.5 (0.1–2.8)	35	30	17.1 (4.0–72.9)
CagA antibodies	Negative	19	70	Ref.	11	19	Ref.	7	49	Ref.
	Positive	40	47	3.5 (1.7–7.1)	4	11	0.6 (0.2–2.5)	34	32	10.9 (3.2–36.9)
Hp-CSAs – CagA antibodies	Hp-CSAs and CagA antibodies negative	15	61	Ref.	10	18	Ref.	4	41	Ref.
	Hp-CSAs or CagA antibodies positive	44	56	3.3 (1.6–6.7)	5	12	0.8 (0.2–2.7)	37	40	9.7 (2.9–32.9)
	Hp-CSAs and CagA antibodies positive	35	32	5.5 (2.3–12.9)	2	8	0.3 (0.03–2.6)	32	22	48.5 (5.8–407.4)

* Odds ratios (ORs) were derived from conditional logistic regression models.

When analysis was limited to 41 cases of non-cardia stomach adenocarcinoma, ORs were 17.1 (95% CI 4.0–72.9) and 10.9 (95% CI 3.2–36.9) among carriers of antibodies to Hp-CSAs and CagA, respectively. The corresponding results for cardia adenocarcinoma were 0.5 (95% CI 0.1–2.8) and 0.6 (95% CI 0.2–2.5), respectively (**Table 2**). Compared with the ‘clean’ reference group, those seronegative for both Hp-CSA and CagA antibodies, subjects seropositive with either Hp-CSA or CagA antibodies had a close to 10-fold increased risk (95% CI 2.9–32.9), and the increased risk further rose to close to 50-fold among those seropositive with both antibodies (95% CI 5.8–407.4). The corresponding results for cardia adenocarcinoma were 0.8 (95% CI 0.2–2.7) and 0.3 (95% CI 0.03–2.6), respectively (**Table 2**).

## Discussion

Although *H. pylori* was classified as a class I carcinogen for stomach cancer by the International Agency for Research on Cancer (IARC) already in 1994 [14], the strength of the association remains uncertain. In an early meta-analysis of 42 studies that were typically of a cross-sectional nature (with *H. pylori* diagnosis at time of cancer diagnosis), the summary odds ratio was only 2.04 [15]. This is probably due to the fact that stomach adenocarcinoma develops late in life (55+), and the histological changes in the stomach during cancer development might lead to spontaneous disappearance of the bacteria. *H. pylori* have been shown to promote the production of inflammatory mediators such as IL-1β and TNF-α, both potent suppressors of stomach juice secretion. The increase in stomach pH may lead to spread of *H. pylori* from the antrum to the corpus, resulting in enhanced inflammation in the mucosa of the corpus, followed by parietal cell destruction and irreversible hypochlorhydria [2], [16].

Already in 1994 a combined analysis of 3 prospective studies found that excess risks increased with increasing time window between sample collection and cancer diagnosis [17]. This finding was corroborated later by a pooled analysis of 12 prospective studies [18], in which a six-fold increased risk for non-cardia adenocarcinoma was found in patients whose samples were collected 10 years or more before cancer diagnosis, while the relative risk within 10 years after serotesting was 2.4. Similarly, in the Alpha-Tocopherol, Beta-Carotene Cancer Prevention (ATBC) prospective cohort study, a close to 8-fold relative risk for non-cardia stomach cancer associated with *H. pylori* infection was reported [19]. *H. pylori* strains containing the *CagA* gene are known to cause more extensive inflammation in the stomach mucosa and antibodies against CagA persist long after eradication [20], [21]. In a Swedish population based case-control study [22], when Hp-CSA-/CagA+ subjects (presumably with “burnt-out” *H. pylori* infection) were removed from the seronegative reference group, subjects seropositive with Hp-CSAs had an OR of 21.0. In particular, the group with Hp-CSA-/CagA+, compared with the reference group seronegative with both tests, has an odds ratio as high as 68.0. This is consistent with a recent Japanese cohort study among middle-aged subjects [23]. There, a more than 100-fold excess risk of stomach cancer was noted in the group seronegative with *H. pylori* antibodies but with a low pepsinogen I:II ratio. Some studies even suggested that *H. pylori* infection might be a necessary cause for non-cardia adenocarcinoma. In a small case-control study performed in Germany, after excluding cases with blood samples collected more than 90 days after gastrectomy, T4 stage, Hp-/CagA+, or borderline IgG results, all the remaining 32 non-cardia stomach adenocarcinoma cases were seropositive with *H. pylori* infection [24]. Similarly, in a small cohort study, in which *H. pylori* status was ascertained by histological examination, rapid urease test, and serologic test, 3% of infected subjects but none of the uninfected developed a stomach cancer, after a mean follow-up time of 7.8 years [25]. Another study conducted in Taiwan with a similar study design also reported none of stomach malignancies detected among the uninfected subjects after a mean follow-up of 6.3 years [26].

In summary, several of the more recent studies suggested that the strength of the *H. pylori*-noncardia stomach adenocarcinoma association might range between a 6-fold to infinite (necessary cause). However, previous studies have various limitations, including small sample size, blood samples collected at old age or at cancer diagnosis, and misclassification of anatomic site of stomach cancer. Our study, based on two large biobanks in Sweden, was able to identify a relative large cohort of subjects, who donated blood samples between ages 16–40, which is the best time window to capture the lifetime occurrence of the infection, as acquisition of the infection almost invariably occurs before adulthood. Further, we retrieved and reviewed medical records for all stomach cancer cases, and managed to clarify the cardia/noncardia origin in all but 3 cases. Our results suggested that the strength of the association between *H. pylori* infection and risk of non-cardia stomach adenocarcinoma was greater than what was reported before in some previous prospective studies among older subjects. However, our data do not provide clear support for the hypothesis that *H. pylori* is a necessary cause of non-cardia stomach adenocarcinoma. Despite small number of cardia adenocarcinoma, our results were consistent with previous report of a null association with *H. pylori* infection in low-risk areas [27].

Despite our efforts to capture *H. pylori* infection status in the best time window, we could not exclude the possibility of misclassification of *H. pylori* infection status. Several lines of evidence support this suspicion, as among the four non-cardia adenocarcinoma cases without any evidence of *H. pylori* infection, one had moderate/severe corpus atrophy at time of serum sampling, suggesting (albeit not proving) previous occurrence of *H. pylori* at some point. Linkage to the Inpatient Register also found that this patient had been hospitalized for viral hepatitis before the date of blood sampling. For the other 3 cases, one had been hospitalized for chronic nephritis before blood sampling, which might also lead to inaccurate measurement of *H. pylori* antibodies. Another limitation of our study is that, due to the age structure of our cohort subjects, most stomach cancer cases were of young age. The association between *H. pylori* infection and stomach cancer might differ as diffuse type is more common among young cases, although previous studies found similar associations for both diffuse and intestinal types [19], [22]. Further, although the cases and controls were matched on age, sex, calendar year of serum collection and biobank, we lacked the information on other potential confounders, like socioeconomic status, smoking and diet, etc. Thus, the possibility of residual confounding cannot not be ruled out.

Our results add confidence to the conclusion that *H. pylori* infection is a much stronger risk factor for non-cardia stomach adenocarcinoma than initially realized, but it is probably not a necessary cause. However, since misclassification of *H. pylori* status may have occurred also in this study, the association might be even stronger than indicated by our data.
